# Structurally robust biological networks

**DOI:** 10.1186/1752-0509-5-74

**Published:** 2011-05-17

**Authors:** Franco Blanchini, Elisa Franco

**Affiliations:** 1 Dipartimento di Matematica ed Informatica, Universitá degli Studi di Udine, Via delle Scienze 206, 33100 Udine, Italy; 2 Division of Engineering and Applied Science, California Institute of Technology, 1200 E. California Blvd. Pasadena, CA 91125, USA

## Abstract

**Background:**

The molecular circuitry of living organisms performs remarkably robust regulatory tasks, despite the often intrinsic variability of its components. A large body of research has in fact highlighted that robustness is often a structural property of biological systems. However, there are few systematic methods to mathematically model and describe structural robustness. With a few exceptions, numerical studies are often the preferred approach to this type of investigation.

**Results:**

In this paper, we propose a framework to analyze robust stability of equilibria in biological networks. We employ Lyapunov and invariant sets theory, focusing on the structure of ordinary differential equation models. Without resorting to extensive numerical simulations, often necessary to explore the behavior of a model in its parameter space, we provide rigorous proofs of robust stability of known bio-molecular networks. Our results are in line with existing literature.

**Conclusions:**

The impact of our results is twofold: on the one hand, we highlight that classical and simple control theory methods are extremely useful to characterize the behavior of biological networks analytically. On the other hand, we are able to demonstrate that some biological networks are robust thanks to their structure and some qualitative properties of the interactions, regardless of the specific values of their parameters.

## Background

The complex biochemistry of living organisms very often outperforms electrical and mechanical devices in terms of adaptability and robustness. Mapping such intricate reaction networks to high level design principles is the goal of systems biology, and it requires an immense collaborative effort among different disciplines, such as physics, mathematics and engineering [1].

The most classical example of robust molecular circuitry is probably given by bacterial chemotaxis [2,3]. The action of the flagellar motor of *E. coli *cells is driven by a cascade of signaling proteins, whose active or inactive state is determined by the presence of nutrient in the environment. Both analysis on a simplified ordinary differential equation (ODE) model [2] and experiments [3] showed how the flagellar motion of *E. coli *presents a robustly stable steady state: steps in the nutrient concentration only temporarily alter the motor equilibrium. Cells are therefore sensitive to nutrient gradients, but always return to their stable motion mode (such property is also referred to as adaptability). Such stable steady state is only a function of the concentrations of the signaling cascade protein components and a few binding rates, and is therefore independent of external inputs. Further analysis also demonstrated how integral feedback is present in the chemotaxis network, and guarantees robustness (perfect adaptation) of the equilibrium [4].

In this work, we are going to ask a simple question: are there biological systems that present structurally stable equilibria, and preserve this property robustly with respect to their specific parameters? This question has been considered before in the literature. For instance, through extensive numerical analysis on three-node networks, the authors of [5] have shown that adaptability of these systems can be investigated solely based on their structure, regardless of the chosen reaction parameters. In [6], through numerical exploration of the Jacobian eigenvalues for two, three and four node networks, the authors isolated a series of interconnections which are stable, robustly with respect to the specific parameters. Such structures also turned out to be the most frequent topologies in existing biological networks databases. Numerical simulation has arguably been the most popular tool to investigate robustness of biological networks [7-12]. Analytical approaches to the study of robustness have been proposed in specific contexts. A series of recent papers [13,14] focused on input/output robustness of ODE models for phosphorylation cascades. In particular, the theory of chemical reaction networks is used in [14] as a powerful tool to demonstrate the property of absolute concentration robustness. Indeed, the so-called deficiency theorems [15] are to date some of the most general results to establish robust stability of a chemical reaction network. Monotonicity is also a structural property that is useful to demonstrate robust dynamic behaviors of a class of biological models [16,17]. Robustness has also been investigated in the context of compartmental models, which are often encountered in biology and chemistry [18].

In this work, we provide a simple and general theoretical tool kit for the analysis bio-molecular systems. Such tools are suitable for the investigation of robust stability by means of Lyapunov and set-invariance methods. Provided that certain standard properties are verified, we demonstrate how a number of well known biological networks are asymptotically stable, robustly with respect to the model parameters. In some cases, we are also able to provide robust bounds on the system performance. Our approach does not require numerical simulation efforts. The contribution of the paper can be summarized as follows.

• The framework we suggest is easy and intuitive for *biologists *to formulate qualitative models without the need of exact mathematical expressions and parameters. We will propose analytical methods that only rely on qualitative interactions between network components.

• The properties that can be derived from such modeling are, consequently, structurally robust because they are not inferred from mathematical formulas arbitrarily chosen to fit data.

• We suggest techniques based on set-invariance and Lyapunov theory, in particular piecewise-linear functions, to show that such models are amenable for robust investigation by *engineers and mathematicians*. Such techniques are believed to be quite effective and promising in dealing with biological robustness [19], [20].

• We consider several models from the literature, reporting the original equations, and rephrasing them in our setup as case studies.

• We show how robust certifications can be given to important properties (some of which have been established based on specific models).

## Methods

### Robustness

We will consider biological dynamical systems which are successfully modeled with ODEs and can be written as:(1)

where *x *is the system state, *u *models external inputs, and both are vectors of appropriate dimensions. Such class of models is appropriate for biological systems where stochasticity and anisotropy can be neglected. We define robustness as follows:

**Definition 1 ***Let **be a class of systems and **be a property pertaining such a class. Given a family **we say that  is robustly verified by *ℱ, *in short robust, if it is satisfied by each element of *ℱ.

Countless examples can be brought about families ℱ and candidate properties. In this work, we will focus on the property of stability, which is an important feature for the equilibria of biological networks [1,6,17]. If we take into account a linear or linearized dynamical system, we can immediately provide an example that clarifies our definition of robustness [21]. Let  be the class of linear differential systems and ℱ the family of second order systems described by

with positive and constant coefficients *a*, *b*, *c*, *d*. Assume . Then we can say that  is robust. The situation is different if we admit that *a*(*t*), *b*(*t*), *c*(*t*), *d*(*t*) can vary with time, yielding a system which is possibly unstable.

If one is interested in the global system behavior, Lyapunov functions are a powerful tool providing sufficient conditions for stability. Given an equilibrium point , any convex function  for  and zero at the origin is a candidate Lyapunov function. If *f*(*x*, *u*) is continuous, and *V *(·) is smooth, then *V *(·) is a Lyapunov function if:

where  is fixed and *κ *(·) is a negative definite function (i.e. *κ *(·) < 0 on all its domain, except for *κ *(0) = 0).

### Non-smooth Lyapunov functions

The concept of Lyapunov derivative can be generalized when the function *V *(·) is non-smooth. For instance, consider the convex function:

where each *V_i_*(·) is smooth and convex, and assume that *V *(·) is positive definite. The set of active functions is never empty and is defined as: . If we define the generalized Lyapunov derivative as:

then the condition for stability becomes:

### Positively invariant sets

We are interested in cases where the trajectories of system (1) remain trapped in bounded sets at all times, therefore behaving consistently with respect to some desired criterion.

We say that a subset  of the state space is positively invariant if  implies that also  for all *t *> 0. The following theorem (which relies on the concept of Lyapunov function) provides a general necessary and sufficient condition for a set to be invariant.

**Theorem 1 ***(Nagumo, 1943) Assume that system *(1) *(for a fixed constant input *) *admits a unique solution. Consider the set:*

*where s_i _are smooth functions, and σ_i _are given constants. Assume that *. *The set of active constraints is *, *and is non-empty only on the boundary of *. *Then the set **is positively invariant if and only if*

For instance, if our constraining functions are linear, *s*^⊤^*x *≤ *σ*, the Nagumo conditions are . We refer the reader to [22] for further details on positively invariant sets; more recent works on this topic are [23] and [24].

### Structural robustness investigation for biological networks

Let us begin with a simple biological example. Consider a protein *x*_1_, which represses the production of an RNA species *x*_2_. In turn, *x*_2 _can be the target of another RNA species *u*_2 _(and form an inactive complex to be degraded) or it can be translated into protein *x*_3_. A standard dynamical model [25] of this process is:(2)

RNA species *x*_2 _determines the production rate of protein *x*_3 _by indexing the corresponding reaction rate as *a*_32_. Following the standard notation in control theory, we assume that the production rate of protein *x*_1 _is driven by some external signal or input *u*_1_, and that RNA *u*_2 _also acts as an external input on RNA *x*_2_. We assume that all the system parameters are positive and bounded scalars. Terms *a_ij _*are first order production rates: species *i *is produced at a rate which is linear in species *j*; *b_ih _*denote in this case first order degradation rates. The term *d*_21_(*x*_1_) is a well known Hill function term [26]. The stability properties of this small network can be immediately assessed: *x*_1 _will converge to its equilibrium . Similarly, , . Regardless of the specific parameter values, and therefore robustly, the system is stable. The equilibrium  could grow unbounded with *u*_1_, however  is always bounded.

We remark that the knowledge of functions *a_ij_x*, *b_ih_x *and *d*(·) *is not necessary at all*: the previous conclusions can be easily derived by the qualitative information that *d*_21 _is strictly decreasing and asymptotically converging to 0, while *b*_11_*x*_1_, *b*_22_*x*_2_, , *a*_32_*x*_2 _and *b*_33_*x*_3 _are increasing.

It is appropriate at this point to outline a series of general assumptions that will be useful in the following analysis.

We will consider a class of biological network models consisting of *n *first order differential equations(3)

where *x_i_*, *i *= 1,..., *n *are the dynamic variables. For the sake of notation simplicity, we are not denoting external inputs with a different symbol. Inputs can be easily included as dynamic variables  which are not affected by other states and have the desired dynamics. The sets  denote the subsets of variables affecting *x_i_*. The different terms in equation (3) are associated with a specific biological and physical meaning. The terms *f_ij_*(· , ·) are associated with production rates of reagents, typically, these functions are assumed polynomial in their arguments; similarly, terms *g_ih _*(· , ·) model degradation or conversion rates and are also likely to be polynomial in practical cases. Finally, terms *c*(·) and *d*(·) are associated with monotonic nonlinear terms, often given by Michaelis-Menten or Hill functions [26]. We assume that system (3) satisfies the following assumptions:

**A1 ***(Smoothness) Functions f_ij _*(· , ·), *g_ih _*(· , ·), *c_is _*(·) *and d_il _*(·) *are unknown nonnegative continuously differentiable functions*.

**A2 ***f_ij _*(*x_i_*, 0) = 0 *and g_ih _*(*x_i_*, 0) = 0, ∀*x*.

**A3 ***Functions f_ij _*(*x_i_*, *x_j_*) *and g_ih _*(*x_i_*, *x_h_*), *are strictly increasing in x_j _and x_h _respectively*.

**A4 ***(Saturation) Functions c_is_*(*x_s_*) *and d_il_*(*x_l_*) *are nonnegative and, respectively, non-decreasing and non-increasing. Moreover c_is_*(∞) > 0 *and d_il_*(0) > 0.

**A5 ***Functions g_ih_*(· , ·) *are null at the lower saturation levels : g_ih_*(0, *x_h_*) = 0, ∀*x_h_*.

In view of the nonnegativity assumptions and Assumption 5, the general model (4) is a nonlinear positive system, according to the next proposition, and its investigation will be restricted to the positive orthant.

**Proposition 1 ***The nonnegative orthant x_i _***≥ **0 *is positively invariant for system *(4).

Given the above assumptions, we can write equation (3) in an equivalent form. First of all, in view of A1-A3, we can write: *f_ij_*(*x_i_*, *x_j_*) = *a*(*x_i_*, *x_j_*)*x_j_*, *g_ih_*(*x_i_*, *x_h_*) = *b*(*x_i_*, *x_h_*)*x_h_*, with

The above expression is always valid due to the smoothness assumption A1 (see [18], Section 2.1).

Additionally, assumption A5 requires that *b_ih_*(0, *x_h_*) = 0, ∀*x_h_*, for *i *≠ *h*. Once we adopt this notation, we can rewrite model (3) as follows:(4)

To simplify the notation, we have considered functions depending on two variables at most. However, we can straightforwardly extend assumptions A1-A5 to multivariate functional terms in equation (3). In turn, the model structure (4) can be easily generalized to include terms as *a*(*x_i_*, *x_j_*, *x_k_*,...), *b*(*x_i_*, *x_j_*, *x_k_*,...), *c*(*x_i_*, *x_j_*, *x_k_*,...), *d*(*x_i_*, *x_j_*, *x_k_*,...).

If we restrict our attention to the general class of models (4), under assumptions A1-A5, we can proceed to successfully analyzing the robust stability properties of several biological network examples.

The structural analysis of system (4) can be greatly facilitated whenever it is legitimate to assume that functions *a*, *b*, *c d *have certain properties. For the reader's convenience, a list of possible properties is given below. Given a general function *f*(*x*):

**P1 ***f *(*x*) = *const ***≥ **0 *is *nonnegative-constant.

**P2 ***f*(*x*) = *const *> 0 *is *positive-constant.

**P3 ***f *(*x*) *is *sigmoidal: *it is non*-*decreasing*, *f*(0) = *f '*(0) = 0, *if *0 <*f*(∞) <**∞ ***and its derivative has a unique maximum point*, *for some *.

**P4 ***f *(*x*) *is *complementary sigmoidal*: it is non*-*increasing*, 0 <*f*(0), *f'*(0) = 0, *f*(∞) = 0 *and its derivative has a unique minimum point. In simple words, f is a CSM function iff f*(0) - *f*(*x*) *is a sigmoidal function*.

**P5 ***f *(*x*) *is *constant-sigmoidal, *the sum of a sigmoid and a positive constant*.

**P6 ***f *(*x*) *is *constant-complementary-sigmoidal, *the sum of a complementary sigmoid and a constant*.

**P7 ***f *(*x*) *is *increasing-asymptotically-constant: *f'*(*x*) > 0, 0 <*f *(∞) <**∞ ***and its derivative is decreasing*.

**P8 ***f *(*x*) *is *decreasing-asymptotically-null: *f'*(*x*) < 0, *f *(∞) = 0 *and its derivative is increasing*.

**P9 ***f *(*x*) *is *decreasing-exactly-null: *f'*(*x*) < 0, *for  and f *(*x*) = 0 *for **for some *.

**P10 ***f *(*x*) *is *increasing-asymptotically-unbounded: *f'*(*x*) > 0, *f *(∞) = + ∞.

**Figure 1:**
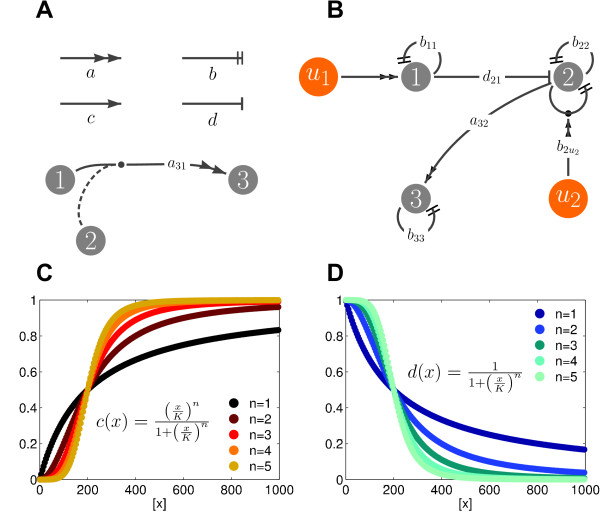
**Graphical representation of biological networks**. A. The arcs associated with the functions *a*, *b*, *c *and *d*. We will use dashed arcs, connecting to arcs of the type *a *and *b *to highlight that the associated function is nonlinearly dependent on a species of the network: in the example above, *a*_31 _= *a*_31_(*x*_2_). B. The graph associated with equations (2); external inputs are represented as orange nodes. C. Examples of sigmoidal functions. D. Examples of complementary sigmoidal functions. In our general model (4), functions *d*(·) and *c*(·) are naturally associated with Hill function terms.

As an example, the terms *d*(·) and *c*(·) are associated with Hill functions, which are *sigmoidal *and *complementary sigmoidal *functions. A graphical sketch of their profile is in Figure 1C and 1D.

### Network graphs

Building a dynamical model for a biological system is often a long and challenging process. For instance, to reveal dynamic interactions among a pool of genes of interest, biologists may need to selectively knockout genes, set up micro RNA assays, or integrate fluorescent reporters in the genome. The data derived from such experiments are often noisy and uncertain, which implies that also the estimated model parameters will be uncertain. However, *qualitative trends *can be reliably assessed in the dynamic or steady state correlation of biological quantities.

Graphical representations of such qualitative trends are often used by biologists, to provide intuition regarding the network main features. We believe that, indeed, such graphs may be useful even to immediately construct models analogous to (3). We propose a specific method to construct such graphs: the biochemical species of the network will be associated to the nodes in the graph, the qualitative relationships between the species will be instead associated with different types of arcs: in particular, the terms of *a*, *b*, *c *and *d *will be represented as arcs having different end-arrows, as shown in Figure 1A. These graphs can be immediately constructed, by knowing the correlation trends among the species of the network, and aid the construction of a dynamical model. For simple networks, this type of graph may provide intuition regarding their behavior and may facilitate their structural robustness analysis. For instance, the graph associated to equations (2) is shown in Figure 1B. Throughout the paper, we will consider similar case studies and use their graph representation as a visual support for the analysis.

**Remark 1 ***In this work, properties such as positivity, monotonicity, boundedness and other functional characteristics are labeled as "qualitative and structural properties"*[27]. *Through such properties, we can draw conclusions on the dynamic behaviors of the considered systems without requiring specific knowledge of parameters and without numerical simulations. However, it is clear that our approach requires more information than other methods, such as boolean networks and other graph-based frameworks*.

### Investigation method

The main objective of this work is to show that, at least for reasonably simple networks, structural robust stability can be investigated with simple analytical methods, without the need for extensive numerical analysis. We will suggest a two stage approach:

• Preliminary screening: establish essential information on the network structure, recognizing which properties (such as P1-P10) pertain to each link.

• Analytical investigation: infer robustness properties based on dynamical systems tools such as Lyapunov theory, set invariance and linearization.

## Results and Discussion

In this section we will analyze five biological networks as case studies. Three of such examples, the L-arabinose, the sRNA and the Lac Operon networks, model the interaction and control of expression of a set of genes. The cAMP and the MAPK pathways are instead signaling networks, namely they represent sets of chemical species interacting for transmission and processing of upstream input signals. These networks are all well-known in the literature, and have been characterized mainly through experimental and numerical methods, although the MAPK pathway, for instance, has been thoroughly analyzed using the theory of monotone systems [17].

We will provide rigorous proofs that these networks are either mono or multi-stable in a robust manner. Such demonstrations rely on Lyapunov functions and invariant sets theory, according to our proposed methodology. In some cases, we are also able to provide bounds on their speed of convergence.

### The L-arabinose network

The arabinose network is analyzed in [28] as an example of feedforward loop. Two genes *araBAD *and *araFGH *are regulated by two transcription factors, AraC and CRP. AraC is a repressor, but turns into an activator when bound to the sugar L-arabinose. CRP is an activator when bound to the inducer cyclic AMP (cAMP), which is produced when cells are starving upon glucose (not produced during growth on glucose). CRP also binds to the *araC *promoter and enhances transcription of AraC, which has a significant basal rate of expression (i.e. it is produced by the cell also in absence of inducer CRP). A very simple model for this network can be derived by defining the state variables *x*_1 _and *x*_2_, respectively the concentrations of the transcription factor AraC and of the output protein araBAD. The concentration of the transcription factor CRP is considered an external input *u*:(5)

where *α*_1_, *α*_2 _are the degradation and dilution rates of *x*_1_, *x*_2 _respectively. The basal production rate of *x*_1 _(AraC) is *p*_1_. The activation pathways are modeled by Hill functions *f *(*u*, *K*) = *u^H ^*/(*K^H ^*+ *u^H ^*), where *H *is the Hill coefficient and *K_ij _*are the activation thresholds. The model can be recast into the general structure (4), which includes model (5) as special case::(6)

where *u *is *nonnegative*-*constant*, *c*_1_, *b*_11 _and *b*_22 _are *positive-constant*, while *c*_1*u*_(*u*) and *c*_2*u*1_(*u*) are *sigmoidal *with respect to *u*, the latter increasing with respect to *x*_1_. The graph representation of this network is in Figure 3A.

For this elementary network the analysis is straightforward. Variable *x*_1 _is not affected by *x*_2_. Since *c*_1*u*_(*u*) is bounded, *x*_1 _is also bounded and converges to an equilibrium point  which is monotonically increasing in *u*. In turn, *x*_2 _is also positive and bounded for any value of *u *and stably converges to a unique equilibrium point , which is a monotonically increasing function of *u *(partially activated by ). The positive term *c*_1 _prevents *x*_1_(*t*) and *x*_2_(*t*) from staying at zero. It is worth remarking that the hierarchical structure of this network greatly facilitates the analysis; equilibria can in fact be iteratively found and their stability properties characterized.

### The sRNA pathway

**Figure 2:**
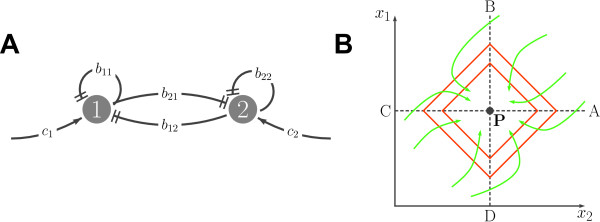
**The sRNA network**. A. The graph associated with the sRNA network B. Sectors, Lyapunov function level curves (orange) and qualitative behavior of the trajectories (green) for the sRNA system

Small regulatory RNAs (sRNA) play a fundamental role in the stress response of many bacteria and eukaryotes. In short, when the organism is subject to a stimulus that threatens the cell survival, certain sRNA species are transcribed and can down-regulate the expression of several other genes. For example, when *E. coli *cells are lacking a source of iron, the sRNA RyhB (normally repressed by the ferric uptake regulator Fur) is expressed and rapidly induces the degradation of at least other 18 RNA species encoding for non-essential proteins which use up Fe molecules. This allows essential iron-dependent pathways to use the low amount of available iron. Quantitative studies of the sRNA pathways have been carried out in [29-31]. Let us define *x*_1 _as the RNA concentration of the species which is targeted by the sRNA and *x*_2 _as the concentration of sRNA. The model often proposed in the literature is:(7)

where *α*_1_, *α*_2 _are the transcription rates of *x*_1 _and *x*_2 _respectively, *β*_1_, *β*_2 _are their degradation rates (turnover), and *k *is the binding rate of the species *x*_1 _and *x*_2_. The formation of the inactive complex *x*_1 _· *x*_2 _corresponds to a depletion of both free molecules of *x*_1 _and *x*_2_. If *α*_1 _<*α*_2 _the pathway successfully suppresses the expression of the non-essential gene encoded by *x*_1_. This model can be embedded in the general family:(8)

by setting *b*_12 _= *kx*_1 _and *b*_21 _= *kx*_2 _(note that *b*_12_(0) = *b*_21_(0) as required). From our list of properties: *c*_1_, *c*_2_, *b*_11 _and *b*_22 _are *positive-constant*; *b*_12_(*x*_1_, *x*_2_) and *b*_21_(*x*_1_, *x*_2_) are *increasing-asymptotically-unbounded *in both variables; and *b*_12_(*x*_1_, *x*_2_)*x*_2 _= *b*_21_(*x*_1_, *x*_2_)*x*_1 _at all times. This network can be represented with the graph in Figure 2A.

The sRNA system is positive, because the nonnegativity Assumptions 1 and 4 are satisfied. The preliminary screening of this network tells us that each variable produce an inhibition control on the other, which increases with the variable itself. In other words *x*_1 _is "less tolerant" to an increase of *x*_2 _if the latter is present in a large amount. This means that the sum *x*_1 _+ *x*_2 _is strongly kept under control. Also the mismatch between the two variables is controlled. ^1 ^To prove stability of the (unique) equilibrium , we will use the 1-norm as Lyapunov function  (see Figure 2B). This choice has a remarkable interpretation: denoting by  and  the sum and the difference of the two variables (referred to the equilibrium) we have

thus the function represents the worst case between the sum and the mismatch.

The following proposition shows that the sRNA pathway is a typical system in which robustness is structurally assured. We report the full demonstration of this proposition, because its steps and the techniques used are a model for the subsequent proofs in this paper.

**Proposition 2 ***The variables of system (8) are bounded for any initial condition x*_1_(0), *x*_2_(0) ≥ 0. *The system admits a unique asymptotically stable equilibrium point **and the convergence is exponential*:(9)

*for some β *> 0 *and any x*_1_(0) ≥ 0, *x*_2_(0) ≥ 0. *Moreover, no oscillations are possible around the equilibrium, in the sense that the condition **or **occurs at most once*.

**Proof **To prove boundedness of the variables we need to show the existence of an invariant set

Proposition 1 guarantees that the positivity constraints are respected. Then we just need to show that the constraint *x*_1 _+ *x*_2 _≤ *κ *cannot be violated for sufficiently large *κ *> 0. The derivative of function *s *(*x*_1_, *x*_2_) = *x*_1 _+ *x*_2 _is

Define *κ *= (*c*_1 _+ *c*_2_)/min {*b*_11_, *b*_22_} then for *s*(*x*_1_, *x*_2_) >*κ *the derivative becomes negative so *s*(*x*_1_, *x*_2_) cannot exceed *κ *(See Theorem 1).

Boundedness of the solution inside a compact set assures the existence of an equilibrium point. Let  be any point in which the following equilibrium conditions holds:(10)

The behavior of the candidate Lyapunov function:

will be examined in the different sectors represented in Figure 2B. Let us start by considering the sector defined by  and  (APB in Figure 2B) for which . In such a sector the Lyapunov derivative is:

where we have subtracted the null terms (10) and where we have exploited the fact that *b*_12_(*x*_1_, *x*_2_)*x*_1 _= *b*_21_(*x*_1_, *x*_2_)*x*_2 _is increasing in both variables. The inequality (CPD in Figure 2B)  can be similarly proved to hold in the sector  and .

Consider the sector defined by  and  (DPA in Figure 2B) for which  in such a sector the Lyapunov derivative is:

Note that in the last step we have added and subtracted the null terms (10). In the opposite sector (BPC in Figure 2B)  and , we can prove that . We just proved that

with *β *= min{*b*_11_, *b*_22_}. This implies (9) and the uniqueness of the equilibrium point.

We finally need to show that there are no oscillations. To this aim we notice that the sectors DPA,  and , and its opposite

CPB,  and , are both positive invariant sets.

We can apply Nagumo's theorem: consider the half-line PA originating in P, where  and . Therefore we have that (again by adding the null term in (10)):

Similarly, on half-line *PD *where  and , by considering (10) we derive

hence the claimed invariance of sector DPA. The proof of the invariance of sector CPB is identical.

**Remark 2 ***Note that the constructed Lyapunov function **does not depend on the system parameters. This fact can be used to prove that if the transcription rates c*_1_*(t) and c*_2_*(t) are time-varying, but bounded, we have convergence to a neighborhood whose amplitude, obviously, depends on the bounds of c*_1_*(t) and c*_2_*(t). It is realistic to assume that the transcription rates vary over time: for instance, if the environmental conditions change, the cell may need to down or up-regulate entire groups of transcripts and therefore increase or decrease c*_2_*(t)*.

The following corollary evidences the positive influence of *c*_2_, which is positive over *x*_2 _and negative over *x*_1_.

**Corollary 1 ***Assume that x*_1_(0), *x*_2_(0) *is at the steady state corresponding to **and *. *Consider the new input **(keeping **). Then the system converges to a new equilibrium with **and *. *There is no undershoot, respectively, overshoot*.

**Proof **The steady state values  and  are respectively monotonically decreasing and increasing functions of *c*_2_. Indeed, consider the steady-state condition

and regard it as a differentiable map (*x*_1_, *x*_2_) → (*c*_1_, *c*_2_). By the uniqueness proved in Proposition 2 the map is invertible. The Jacobian of the inverse map is the inverse of the Jacobian

where  (keep in mind that *b*_21_(*x*_1_, *x*_2_)*x*_1 _= *b*_12_(*x*_1_, *x*_2_)*x*_2_). The sign of the entries in the second column are negative and positive respectively, therefore, the steady-state values  and  are decreasing and increasing functions of *c*_2_.

The absence of overshoot and undershoot is an immediate consequence of the invariance of the sector  and  previously proved.

Obviously, decreasing *c*_2 _increases *x*_1 _and decreases *x*_2 _and the same holds if we commute 1 and 2. It is worth noting that the same conclusions regarding the lack of multistability and oscillations for the sRNA pathway may be reached by qualitative analysis of the system's nullclines.

### The cAMP dependent pathway

**Figure 3:**
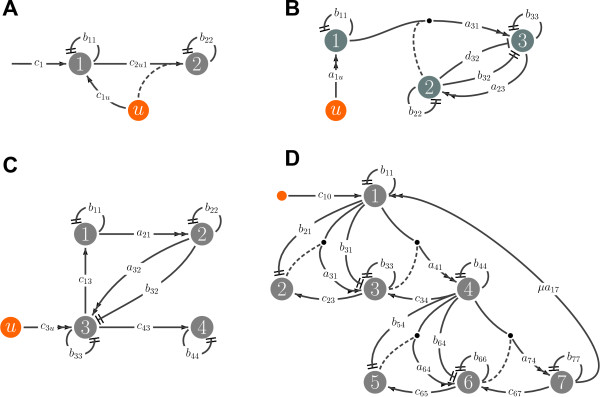
**Graphs associated with case studies**. A. The graph associated with the L-arabinose network, external inputs are represented as orange nodes. B. The graph associated with the cAMP pathway. C. The graph associated with the lac Operon network. D. The graph associated with the MAPK signaling pathway.

The cyclic adenosine monophosphate (cAMP) pathway can activate enzymes and regulate gene expression based on sensing of extracellular signals. Such signals are sensed by the G protein-coupled receptors on the cell membrane. When a receptor is activated by its extracellular ligand, a series of conformational changes are induced in the receptor and in the attached intracellular G protein complex; the latter activates adenylyl cyclase, which catalyzes the conversion of ATP in cAMP. In yeast, cAMP causes the activation of the protein kinase A (PKA), which in turn regulates the cell growth, metabolism and stress response. Stochastic models are usually proposed for numerical analysis of the cAMP pathway. However, the cAMP pathway components in yeast are present in high numbers and a deterministic modeling approach is adopted in [31]. In such paper, the pathway is decomposed in several modules, here we consider the simplified cAMP Model A, which focuses on the parts of the pathway best characterized in the literature:(11)

where *x*_1 _is the concentration of active G protein, *x*_2 _is the concentration of active PKA protein, *x*_3 _is the concentration of cAMP and *u *is the concentration of glucose input to the network. The parameters  and  model the "feedback" effect introduced by two phosphodiesterases (Pde1p and Pde2p) on the cAMP concentration. The numerical analysis in [31] typically shows that the cAMP concentration (*x*_3_) responds with a large overshoot to steps in the glucose (*u*, input) concentration. We will analytically explore the dynamic behavior of *x*_3_, showing that under certain assumptions, a bounded overshoot is a robust characteristic in the system. The parameters *k_F _*and *k_R _*are forward and reverse reaction rates for the formation of active *x*_1 _and *x*_2_. Mass conservation allows to express the active protein amounts as a function of the total number of molecules, . The nonlinear expressions in equation *x*_3 _are derived by Michaelis-Menten enzymatic steps. We can re-write the above equations according to the general model (4):(12)

where *u *is the external signal and where *b*_23 _= 0 for *x*_2 _= 0 and *b*_32 _= 0 for *x*_3 _= 0. A qualitative graphical representation of this network is in Figure 3B.

Our preliminary analysis allows us to assume: *a*_1*u*_, *a*_23_: *decreasing-exactly-null *with threshold values  and ; *d*_32_, *a*_31_: *decreasing-asymptotically-null*, *b*_32 _and *g*_33 _= *b*_33_(*x*_3_)*x*_3_: *increasing-asymptotically-constant; b*_11_, *b*_22 _*are positive-constant*.

It is immediate to notice that for constant *u*, *x*_1 _robustly converges asymptotically to its equilibrium value such that

The solution  of the previous equation is uniquely defined for each *u *since the function *ξ*^-1^(*x*_1_) on the right is strictly increasing and grows to infinity, precisely . Biologically, this means that external glucose signals are mapped to internal active G-protein concentration with a bijection, before saturating.

Also we have to note that the model is consistent with mass conservation: since *a*_1*u*_(*x*_1_) and *a*_23_(*x*_2_) are zero above the thresholds  and , we have that  and  for  and , respectively; therefore we assume , , for all *t *≥ 0.

**Proposition 3 ***There exists an equilibrium for system *(12) *if and only if*(13)

*where **as previously defined *. *All the equilibrium values *, *and **are increasing functions of u. If condition (13) is satisfied *, *the equilibrium is unique and locally stable*.

The previous proposition assures only local stability, but this result can be extended to global stability. To this aim, we will assume that *x*_1 _is at its equilibrium value . Furthermore, under a suitable condition a performance bound on the transient values of *x*_3_(*t*) can be given.

**Proposition 4 ***Assume that x*_1 _*has reached its steady state *. *Then, the unique equilibrium point is globally attractive for any initial condition x*_2_(0), *x*_3_(0) ≥ 0. *Moreover, assume that*(14)

*then we can give the following bound for the transient of x*_3_(*t*)(15)

The proof can be found in Section S{II of the Additional File 1.

**Remark 3 ***The condition (14) has the following interpretation. It basically states that the inhibiting term b*_33_(*x*_3_)*x*_3 _*at "full force" (x*_3 _*suitably large) dominates the activating term d*_32_(*x*_2_) + *a*_31_(*x*_2_)*ξ when x*_2 _*is small. Note that, indeed, the feedback terms modulated by the two phosphodiesterases act in a complementary manner*, *in order to maintain a bounded concentration of cAMP in the cell*.

**Remark 4 ***The system, even if initialized with small values x_2_*(0) *and x*_3_(0), *may exhibit a spike of cAMP x*_3 _*which is bounded by (15), if condition (14) is satisfied. If x_3_(0) is small, then the bound is d*_32_(0) + *a*_31_(0)*ξ *(*u*): *the amplitude of the spike is, in general, an increasing function of the glucose concentration u. If condition (14) fails, then (see Figure S2 in the Additional File) the spike of x*_3_*(t) can be arbitrarily large; thus condition (14) can be seen as a threshold*.

### The *Lac *operon

This genetic network was originally studied by Monod and Jacob [33]. The natural nutrient for *E. coli *bacterial cells is glucose, which is metabolized by enzymes normally produced by the bacteria. When glucose is absent, but the allolactose inducer is present in their environment, *E. coli *activates a set of genes that will regulate the lactose intake and breakdown. In particular, the cells start producing a permease protein, which binds to the cell membrane and increases the inflow of lactose; and cells also start producing the *β*-galactosidase protein, which converts lactose in allolactose.

In this paper we will consider the deterministic model proposed in [34]. This simple model does not capture the stochasticity of this genetic circuit, but it does explain the bimodal behavior of the system. Such behavior is observable experimentally: within the same population, the operon can be either induced or uninduced. Our analysis shows that for low or high intracellular inducer concentrations, the system is monostable and respectively reaches an uninduced or induced equilibrium; however, at intermediate inducer concentrations the system becomes multi-stable.

The state variables of the ODE model we will study are the concentration of nonfunctional permease protein *x*_1_; the concentration of functional permease protein *x*_2_; the concentration of inducer (allolactose) inside the cell *x*_3_, and the concentration of *β*-galactosidase *x*_4_, a quantity that can be experimentally measured. The concentration of inducer external to the cell is here denoted as an input function *u*.(16)

where *β*_1_, *β*_2_, *δ*_1_, *δ*_2_, *δ*_3 _and *γ *are constants and *f_i _*are functions that are experimentally measurable. In particular, at low inducer concentrations, , where *k_i_*'s are constant; at high *x*_3 _concentrations *f*_1 _saturates. The functions *f*_2 _and *f*_3 _are assumed to depend hyperbolically on their arguments. According to the proposed setup, the previous equations can rewritten as follows:(17)

where *c*_13_(*x*_3_) = *f*_1_(*x*_3_), *b*_11 _= *δ*_1_, *a*_21 _= *β*_1_, *b*_22 _= *δ*_2_, *a*_32_(*u*) = *f*_2_(*u*) =, *b*_32_(*x*_3_) = *f*_3_(*x*_3_), *c*_3*u *_= *β*_2_, *b*_33 _= *δ*_3_, *c*_43_(*x*_3_) = *γ f*_1_(*x*_3_) and *b*_44 _= *δ*_4_. This corresponds to the network in Figure 3C.

From our preliminary analysis step: *c*_13 _is *constant-sigmoidal*, *a*_32_(*u*) and *b*_32_(*x*_3_) are *increasing-asymptotically-constant*, and the remaining functions *a*_21_, *b*_11_, *b*_22 _and b_33 _are *positive-constant*.

We can start to study this network without any specific knowledge of the parameters in equations (17). First of all, as evident in Figure 3C, note that the *β*-galactosidase concentration *x*_4 _does not affect any other chemical species: therefore, the fourth equation can be considered separately. As long as the inducer concentration of *x*_3 _within the cell reaches an equilibrium , *x*_4 _converges to . Therefore, we can restrict our attention to the first three equations; this is consistent with the model proposed in [35,36]. From now on we will consider this reduced model (see Section S-III of the Additional File), neglecting the linear term *c*_3*u*_*u *as in [35,36]. We will not introduce delays in our model, as done in [37]. Our preliminary screening also shows that the evolution of this system is necessarily bounded. Indeed *x*_1 _receives a bounded signal from *x*_3 _and the degradation term -*b*_11_*x*_1 _keeps *x*_1 _bounded. In turn, *x*_2 _remains bounded. The inducer concentration *x*_3 _receives a bounded signal form *u *and *x*_2_; therefore *x*_3 _stays bounded as well, being both *a*_32_(*u*) and *b*_32_(*x*_3_) bounded.

The following proposition evidences that fundamental results can be established starting from our general framework. These results are consistent with the findings in [36], whose analysis relies on assuming Hill-type functions in the model.

**Proposition 5 ***For any functional terms in Equations 17, satisfying the general assumptions formulated above, the system admits a unique equilibrium for large u *> 0 *or small u *> 0.

*For some chioces of such functional terms, the system may have multiple positive equilibria x^A^*, *x^B^*, *x^C ^*,... ∈ IR^3 ^*(typically three) for intermediate values of u. If multiple equilibria exist, then they are ordered in the sense that x^A ^*≤ *x^B ^*≤ *x^C ^*... *where the inequality has to be considered componentwise. If the equilibria are all distinct, then they are alternatively stable and unstable. In the case of three equilibria, x^A^*, *x^B^*, *x^C ^they are stable, unstable and stable, respectively. Finally, given any equilibrium point, the positive and negative cones x *≤ x* *and × *≥ x* *are positively invariant*.

The proof is given in Section S-III of the Additional File. The cone-invariance property implies that the state variables cannot exhibit oscillations around their equilibria. For instance, if *x^A ^*is the first (hence stable) equilibrium, given any initial condition upper bounded by *x^A ^*(*x*(0) *x^A^*) in the domain of attraction, the convergence to *x^A ^*has no overshoot (and if *x*(0) ≥ *x^A ^*there is no undershoot).

**Remark 5 ***It is interesting to notice that, due to the competition between terms a*_32 _*and b*_32_, *the considered Lac Operon model is not a monotone system according to the definition in *[16], *where a different model was considered*.

### MAPK signaling pathway

Mitogen-activated protein (MAP) kinases are proteins that respond to the binding of growth factors to cell surface receptors. The pathway consists of three enzymes, MAP kinase, MAP kinase kinase (MAP2K) and MAP kinase kinase kinase (MAP3K) that are activated in series. By activation or phosphorylation, we mean the addition of a phosphate group to the target protein. Extracellular signals can activate MAP3K, which in turn phosphorylates MAP2K at two different sites; in the last round, MAP2K phosphorylates MAPK at two different sites. The MAP kinase signaling cascade can transduce a variety of growth factor signals, and has been evolutionary conserved from yeast to mammals.

Several experimental studies have highlighted the presence of feedback loops in this pathway, which result in different dynamic properties. This work will focus on a specific positive-feedback topology, where doubly-phosphorylated MAPK has an activation effect on MAP3K. Such positive feedback has been extensively studied in the literature, since the biochemical analysis of Huang and Ferrell [37,38] on the MAPK cascade found in *Xenopus *oocytes. In this type of cells, Mos (MAP3K) can activate MEK (MAP2K) through phosphorylation of two residues (converting unphosphorylated MEK to monophosphorylated MEK-P and then bisphosphorylated MEK-PP). Active MEK then phosphorylates p42 (MAPK) at two residues. Active p42 can then promote Mos synthesis, completing the closed positive-feedback loop.

The presence of such positive feedback in the MAPK cascade has been linked to a bistable behavior: the switch between two stable equilibria in *Xenopus *oocytes denotes the transition between the immature and mature state. A standard ODE model for the cascade is proposed in [17], where the authors demonstrate bi-stability of the system by applying the general theory of monotone systems. We adopt such model, which is reported below:(18)

where *x *is concentration of Mos (MAP3K), *y*_1 _is the concentration of unphosphorylated MEK (MAP2K), *y*_2 _is the concentration of phosphorilated MEK-P, *y*_3 _is the concentration of MEK-PP, *z*_1_, *z*_2 _and *z*_3 _are respectively the concentrations of unphosphorylated, phosphorylated and doubly-phosphorylated p42 (MAPK). Finally, *u *is the input to the system.

While bi-stability may occur due to other phenomena, such as multisite phosphorylation [39], rather than due to feedback loops, a large body of literature focuses on bi-stability induced by the positive-feedback in the Huang-Ferrel model in *Xenopus *[40,41] reported above. In [37] the feedback *f *(*u*) was characterized, through *in vitro *studies, as an activating Hill-function with high cooperativity. In [17] instead, *f *(*u*) was assumed to be a first order linear term in the concentration of active MAP3K, *x*_7_. In Proposition 6, we will explore the effects of different qualitative functional assumptions on the feedback loop dynamics *f *(*u*). We will highlight that the system loses its well-known bi-stability not only in the absence of feedback, but also when the feedback becomes unbounded. An unbounded positive feedback would be caused, for instance, by an autocatalytic process of MAP3K activation, mediated by active MAPK. We choose to rewrite the above model as follows:(19)

The term *μx*_7 _introduces the positive feedback loop and represents a key parameter for the analysis to follow. A preliminary screening of the system immediately highlights the following properties. Function *b*_11_(*x*_1_)*x*_1_, functions *c*_23_(*x*_3_), *b*_21_(*x*_2_), *a*_41_(*x*_3_) and *b*_44_(*x*_4_)*x*_4_, functions *c*_56_(*x*_6_), *b*_54_(*x*_5_), *a*_74_(*x*_6_) and *b*_77_(*x*_7_)*x*_7 _are *increasing-asymptotically-constant*. Moreover, *a*_31_(*x*_2_) = *b*_21_(*x*_2_), *c*_34_(*x*_4_) = *b*_44_(*x*_4_)*x*_4_, *b*_31_(*x*_3_) = *a*_41_(*x*_3_), *b*_33_(*x*_3_)*x*_3 _= *c*_23_(*x*_3_) and *a*_64_(*x*_5_) = *b*_54_(*x*_5_), *c*_67_(*x*_7_) = *b*_77_(*x*_7_)*x*_7_, *b*_64_(*x*_6_) = *a*_74_(*x*_6_), *b*_66_(*x*_6_)*x*_6 _= *c*_56_(*x*_6_). We assume *c*_10 _to be a *positive-constant*.

The graph in Figure 3D can be partitioned considering three aggregates of variables, precisely {*x*_1_}, Σ_234 _= (*x*_2_, *x*_3_, *x*_4_) and Σ _567 _= {*x*_5_, *x*_6_, *x*_7_}. Signal *x*_1 _is the only input for Σ_234_, signal *x*_4 _is the only input for Σ_567_. Then *x*_7 _is fed back to the first subsystems by arc *a*_17_. Without the positive feedback loop, we will demonstrate that the system is a pure stable cascade. Note also that Σ_234 _and Σ_567 _can be reduced since  and  and therefore the following sums are constant(20)

with *k *≐ *x*_2_(0) + *x*_3_(0) + *x*_4_(0) and *h *≐ *x*_5_(0) + *x*_6_(0) + *x*_7_(0). Since *x_i _*≥ 0, all the variables but *x*_1 _are bounded. The system can be studied by removing variables *x*_3 _= *k *- *x*_2 _- x_4 _and *x*_6 _= *h *- *x*_5 _- *x*_7_. We must assume that  otherwise no equilibrium is possible. The following result is proved in Section S-IV of the Additional File.

**Proposition 6 ***For μ *= 0 *the system admits a unique globally asymptotically stable equilibrium*.

*For μ *> 0, *the system may have multiple equilibria, for specific choices of the involved functions a*, *b*, *c*.

*For μ *> 0 *suitably large and a*_17_(*x*_1_) *lower bounded by a positive number, then the system has no equilibria*.

*For μ *> 0 *suitably bounded and a*_17_(*x*_1_) *increasing, or non-decreasing, and bounded, if multiple simple*^2 ^*equilibria exist, then such equilibria are alternatively stable and unstable. In the special case of three equilibria, then the system is bistable*.

*For μ *> 0 *suitably bounded and a*_17_(*x*_1_*) increasing asymptotically unbounded, then the number of equilibria is necessarily even (typically *0 *or *2). Moreover, if we assume that there exists μ* > 0 *such that the system admits two distinct equilibria for any *0 <*μ *≤ μ*, *then one is stable, while the other is unstable*.

The proof of this last proposition also shows that multiple equilibria *x^A^*, *x^B^*,... have a partial order:  while  and  have the reverse order  and 

**Remark 6 ***The simplest case of constant a*_17 _*has been fully developed in *[17]^3 ^*and *[16], *and it turns out that the system may exhibit bi-stability for suitable values of the feedback strength μ. Here we show that, for constant a*_17_, *bi-stability is actually a robust property. Our results are consistent with the fact that the MAPK cascade is a monotone system and some of them could be demonstrated with the same tools used in *[16,17]. *With respect to such literature, our contribution is that of inferring properties such as number of equilibria and mono or bi-stability starting from qualitative assumptions on the dynamics of the model, without invoking monotonicity*.

**Remark 7 ***Finally, it is necessary to remark that our results on the MAPK pathway robust behaviors hold true given the model (19) and its structure. Other work in the literature shows that feedback loops are not required to achieve a bistable behavior in the MAPK cascade *[38], *when the dual phosphorylation and de-phosphorylation cycles are non-processive (i.e. sites can be phosphorylated/de-phosphorylation independently) and distributed (i.e. the enzyme responsible for phosphorylation/de-phosphorylation is competitively used in the two steps)*.

## Conclusions

A property is structurally robust if it is satisfied by a class of systems of a given structure, regardless the choice of specific expressions adopted and of the parameter values in the model. We have considered five relevant biological examples and proposed to capture their dynamics with parameter-free, qualitative models. We have shown that specific robust properties of such models can be assessed by means of solid theoretical tools based on Lyapunov methods, set-invariance theory and matrix theory. Robustness is often tested through simulations, at the price of exhaustive campaigns of numerical trials and, more importantly, with no theoretical guarantee of robustness. We are far from claiming that numerical simulation is useless. It it important, for instance, to falsify "robustness conjectures" by finding suitable numerical counterexamples. Furthermore, for very complex systems in which analytic tools can fail, simulation appears be the last resort. Indeed a limit of the considered theoretical investigation is that its systematic application to more complex cases is challenging. However, the set of techniques we employed can be successfully used to study a large class of simple systems, and are in general suitable for the analytical investigation of structural robustness of biological networks, complementary to simulations and experiments.

## Authors' contributions

FB and EF performed research and wrote the paper.

## Notes

^1^The concentration mismatch is more "softly" controlled, since the derivative of the difference  is not influenced by the nonlinear term *b*_12_(*x*_1_, *x*_2_)*x*_2 _= *b*_21_(*x*_1_, *x*_2_)*x*_1_.

^2^I.e. the nullclines have no common tangent lines.

^3^Cf. the erratum: http://www.math.rutgers.edu/~sontag/FTPDIR/angeli-ferrell-sontag-pnas04-errata. txt and [42].

## Supplementary Material

Additional file 1 **One additional file includes the proofs for Propositions 3, 4, 5 and 6 in the main paper**. Click here for file
