# Supplementary material for: Structurally robust biological networks
Source: BMC Syst Biol. 2011 May 17;5:74. doi: 10.1186/1752-0509-5-74 (PMC3125314; doi:10.1186/1752-0509-5-74)
Supplement: Additional file 1 — One additional file includes the proofs for Propositions 3, 4, 5 and 6 in the main paper. [file 1752-0509-5-74-S1.PDF]

Structurally robust biological networks
Additional File

Franco Blanchini[1]and Elisa Franco[2*]

[1] Dipartimento di Matematica ed Informatica, Università degli Studi di Udine, Via delle Scienze 206, 33100 Udine, Italy
[2] Division of Engineering and Applied Science, California Institute of Technology, 1200 E. California Blvd. Pasadena, CA 91125, USA.

Email: Franco Blanchini - blanchini@uniud.it; Elisa Franco - elisaf@caltech.edu;

*Corresponding author

This additional file includes the proofs to Propositions 3, 4, 5 and 6 in the main paper.

S–I Proof of Proposition 3 (equilibrium of the cAMP signaling pathway)

Consider our model for the cAMP pathway, given in equations (12) in the main text:

$$\begin{aligned}\dot{x}_1 &= a_{1u}(x_1)u - b_{11}x_1,\\ \dot{x}_2 &= a_{23}(x_2)x_3 - b_{22}x_2,\\ \dot{x}_3 &= d_{32}(x_2) + a_{31}(x_2)x_1 - b_{32}(x_3)x_2 - b_{33}(x_3)x_3.\end{aligned}$$

Proposition 3: *There exists an equilibrium for system* (12) *if and only if*

$$d_{32}(x_2^{tot}) + a_{31}(x_2^{tot})\bar{x}_1 < \lim_{x_3\to\infty}\left[b_{32}(x_3)x_2^{tot} + b_{33}(x_3)x_3\right], \tag{S1}$$

where $\bar{x}_1 = \xi(u)$. All the equilibrium values $\bar{x}_1 = \xi(u)$, $\bar{x}_2$ and $\bar{x}_3$ are increasing functions of u. If condition (S1) is satisfied, the equilibrium is unique and locally stable.

Proof: Given the steady state value for $\bar{x}_1 = \xi(u)$, the equilibrium values for x_2 and x_3 are determined by the following equations:

$$\begin{aligned}x_3 &= \frac{b_{22}x_2}{a_{23}(x_2)} \doteq \varphi(x_2),\\ 0 &= d_{32}(x_2) + a_{31}(x_2)\xi - b_{32}(x_3)x_2 - b_{33}(x_3)x_3 \doteq \psi(x_2, x_3).\end{aligned}$$

The curve $x_3 = \varphi(x_2)$ has a vertical asymptote corresponding to x_2^{tot}. Assume condition (S1) is satisfied.

This means that the equation $\psi(x_2^{tot}, x_3) = 0$, or equivalently

$$d_{32}(x_2^{tot}) + a_{31}(x_2^{tot})\xi = b_{32}(x_3)x_2^{tot} + b_{33}(x_3)x_3,$$

has a solution because the term $b_{32}(x_3)x_2^{tot} + b_{33}(x_3)x_3$ is monotonically increasing from zero to its limit value which exceeds the left hand term.

By continuity, $\psi(x_2, x_3) = 0$ is solvable for x_2 sufficiently close to x_2^{tot}. This means that the curve $\psi(x_2, x_3) = 0$ can be extended on the left of the asymptote, namely for $x_2 < x_2^{tot}$.

Since it is not possible to derive explicitly x_3 as a function of x_2 we need to resort to the implicit function theorem. As a first step, we notice that $\partial\psi(x_2, x_3)/\partial x_3$ never vanishes for positive values of the variables. Then it is possible to define the implicit function $x_3(x_2)$. The derivative of such function is

$$\frac{dx_3}{dx_2} = -\frac{\partial\psi(x_2, x_3)/\partial x_2}{\partial\psi(x_2, x_3)/\partial x_3} = \frac{d'_{23} + a'_{31}\xi - b_{32}}{b'_{32}x_2 + (b_{33}(x_3)x_3)'} < 0.$$

(Note that $d'_{23}, a'_{31} \leq 0$, $b'_{32} > 0$, $(b_{33}(x_3)x_3)' > 0$). Therefore $x_3(x_2)$ is a decreasing function and the left extension of such a curve can be interrupted either when it encounters the x_3–axis i.e. $x_2 = 0$ (as in Figure S1) or when it encounters a vertical asymptote which is on the left of x_2^{tot} (as in Figure S2).

On the other hand $\varphi(x_2)$ is strictly increasing, originating from 0 and defined over $[0, x_2^{tot})$ which in turn implies that under the condition (S1) the two curves have an unique intersection (the point P in Figure S1).

We can easily prove that the steady state values are monotonic functions of the input u. It is immediate that $\bar{x}_1 = \xi(u)$ is an increasing function of u. In turn, $\bar{x}_2$ and $\bar{x}_3$ are increasing functions of x_1: this can be verified by substituting $x_3 = \varphi(x_2)$ in $\psi(x_1, x_2) = \psi(x_2, \varphi(x_2))$ to achieve

$$d_{32}(x_2) + a_{31}(x_2)x_1 - b_{32}(\varphi(x_2))\varphi(x_2) - b_{33}(\varphi(x_2))\varphi(x_2) = 0.$$

Using the implicit function theorem again, one can see that

$$\frac{dx_2}{dx_1} = -\frac{a_{31}(x_2)}{d'_{32}(x_2) + a'_{31}(x_2)\xi - (b_{32}(\varphi)\varphi)' - (b_{33}(\varphi)\varphi)'} > 0.$$

The monotonicity of x_3 is true in view of the fact that $x_3 = \varphi(x_2)$ is monotonic.

Stability of the equilibrium can be easily inferred by the Jacobian matrix

$$J = \begin{bmatrix} a'_{1u}\bar{u} - b_{11} & 0 & 0 \\ 0 & -[a'_{23}x_3 + b_{22}] & a_{23} \\ a_{31} & [d'_{32} + a'_{31}x_1 - b_{32}] & -[b'_{32} + (b_{33}x_3)'] \end{bmatrix} \approx \begin{bmatrix} - & 0 & 0 \\ 0 & - & + \\ + & - & - \end{bmatrix},$$

where $\approx$ denotes the sign pattern. Any matrix with such sign pattern has eigenvalues with negative real parts. □

S–II Proof of Proposition 4 (stability of the cAMP signaling pathway)

Proposition 4: *Assume that x_1 in system (12) has reached its steady state $\bar{x}_1$. Then, the unique equilibrium point is globally attractive for any initial condition $x_2(0), x_3(0) \geq 0$. Moreover, assume that*

$$l_3 \doteq \lim_{x_3 \to \infty} b_{33}(x_3)x_3 > d_{32}(0) + a_{31}(0)\xi, \tag{S2}$$

then we can give the following bound for the transient of $x_3(t)$

$$x_3(t) \leq \max\{x_3(0), d_{32}(0) + a_{31}(0)\xi\}. \tag{S3}$$

Proof: We will prove the proposition under the condition (S2) and sketch the proof in general.

Under condition (S2) the curve $\psi(x_2, x_3) = 0$ (derived by setting $\dot{x}_3$=0) intersects the x_3 axis (point B in Figure S1). Indeed for $x_2 = 0$ we have the equation $0 = d_{32}(0) + a_{31}(0)\xi - b_{33}(x_3)x_3$ which is satisfied for some x_3 in view of (S2). For any other value of $x_2 > 0$ the equation $\psi(x_2, x_3) = 0$ is

$$0 = d_{32}(x_2) + a_{31}(x_2)\xi - b_{32}(x_3)x_2 - b_{33}(x_3)x_3,$$

which is obviously solvable in x_3 since $d_{32}(x_2) + a_{31}(x_2)\xi < d_{32}(0) + a_{31}(0)\xi$.

We have already proved that the function $\psi(x_2, x_3) = 0$ and the curve defined by $x_3 = \varphi(x_2)$ intersect in a single equilibrium point. In the following we will show that any trajectory starting from any initial condition is bounded. As sketched in Figure S1, consider any rectangle AFCE large enough to include (if

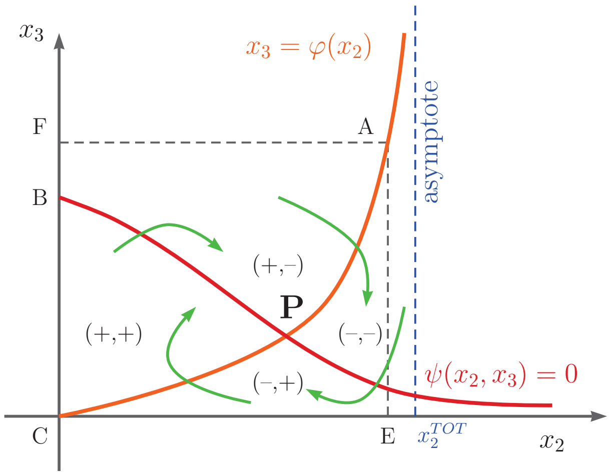

Figure S1: Phase plane for the cAMP system

possible) the initial condition, with a vertex A on the curve $\dot{x}_2 = 0$, namely $\varphi(x_2) = x_3$. Note that the two

curves $\psi(x_2, x_3) = 0$ and $\varphi(x_2) = 0$ define four regions in which the derivative sign (sign$\dot{x}_2$, sign$\dot{x}_3$) are $(--)$, $(+-)$, $(++)$ and $(-+)$ starting from region APD counterclockwise.

Take the rectangle large enough in such a way that F is above B, the intersection of $\psi(x_2, x_3) = 0$ with the x_3 axis and above $x(0)$. Consider the sign of the derivatives: on the line FA we have $\dot{x}_3 < 0$, while on AE we have $\dot{x}_2 < 0$. This means that the rectangle AFCE is positively invariant. Note that an initial condition on the right of the asymptote of the curve $x_3 = \varphi(x_2)$ (Figure S1) is not captured by this construction. This is not an issue, since on the right the asymptote have $\dot{x}_2 < 0$ and $\dot{x}_3 < 0$, which means that the trajectory reaches the rectangle crossing the segment AE. So any trajectory eventually reaches an invariant rectangle. This system is planar, therefore there are two possibilities: either the trajectory reaches a limit circle or it reaches the equilibrium point (see, for instance, [1], Theorem 2.2). To show that no limit cycles exist, consider the divergence of the vector field

$$\frac{\partial f_2}{\partial x_2} + \frac{\partial f_3}{\partial x_3} = a'_{23}(x_2)x_3 - b_{22} - b'_{32}(x_2) - (b_{33}(x_3))' < 0.$$

In view of the Poincare–Bendixson theorem (see [1], Theorem 2.3) no limit cycles may exist, therefore any solution converges to the equilibrium.

The bound (S3) can be easily proved by selecting point A in Figure S1, and in turn the bounding segment FA, to fall on the curve at a height equal to $\max\{x_3(0), d_{32}(0) + a_{31}(0)\xi\}$.

If (S2) does not hold, we can intuitively explain why convergence is still assured with the aid of Figure S2.

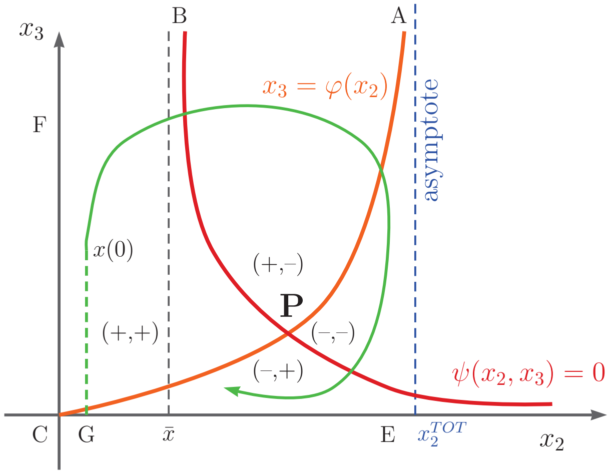

Figure S2: Generalization of the phase plane represented in Figure S1

In general the curve $\psi(x_2, x_3) = 0$, may have a vertical asymptote. Consider an initial condition in the sector defined by the curve branch CPB. Here both derivatives are positive meaning that any trajectory

reaches the branch PB (possibly in P). In the sector defined by the branch BPA, we have $\dot{x}_2 > 0$ and $\dot{x}_3 < 0$, which means that the branch PA is reached (again possibly in P). In the same way one can see that the sectors are encountered clockwise. It is not difficult (but tedious) to prove that the curve will remain trapped in the region defined by the curve itself and the additional segment $x(0)$G (because $\dot{x}_2 > 0$ in the sector CPB). This proves boundedness of the solution. However no bounds for the overshoot can be given, since the intersection of the trajectory with the PB branch can be arbitrarily high. Similar considerations can be brought to prove boundedness if the initial condition is elsewhere. □

S–III Proof of Proposition 5 (properties of the Lac Operon equilibria)

Consider the following model for the Lac network:

$$\begin{aligned} \frac{dx_1}{dt} &= c_{13}(x_3) - b_{11}x_1, \\ \frac{dx_2}{dt} &= a_{21}x_1 - b_{22}x_2 \\ \frac{dx_3}{dt} &= a_{32}(u)x_2 - b_{32}(x_3)x_2 - b_{33}x_3. \end{aligned} \tag{S4}$$

Proposition 5: *For large $u > 0$ or small $u > 0$ the system admits a unique equilibrium. The system may have multiple equilibria $x^A, x^B, x^C, \dots \in \mathbb{R}^3$ (typically three) for intermediate values. If multiple equilibria exist, then they are ordered in the sense that $x^A \le x^B \le x^C \dots$ where the inequality has to be considered componentwise. If the equilibria are all distinct, then they are alternatively stable and unstable. In the case of three equilibria, x^A, x^B, x^C they are stable, unstable and stable, respectively. Finally, given any equilibrium point, the positive and negative cones $x \le x^*$ and $x \ge x^*$ are positively invariant.*

Proof: The equilibrium conditions for system (S4) are $c_{13}(x_3) - b_{11}x_1 = 0$, $a_{21}x_1 - b_{22}x_2 = 0$ and $a_{32}(u)x_2 - b_{32}(x_3)x_2 - b_{33}x_3 = 0$. Defining $\varphi(x_3) \doteq a_{21}c_{13}(x_3)/(b_{11}b_{22})$ we can derive the following equation:

$$\psi(x_3) \doteq b_{32}(x_3) + \frac{b_{33}x_3}{\varphi(x_3)} = a_{32}(u),$$

or equivalently

$$\phi(x_3, u) \doteq b_{33}x_3 + b_{32}(x_3)\varphi(x_3) - a_{32}(u)\varphi(x_3) = 0,$$

whose roots correspond to the equilibria of system (S4). The qualitative behavior of the functions $\psi(x_3)$ and $\phi(x_3, u)$ is reported in Figure S3. For $x_3 = 0$, $\psi(x_3) = 0$, while $\phi(x_3, u) < 0$ because $\varphi(0)$ is positive

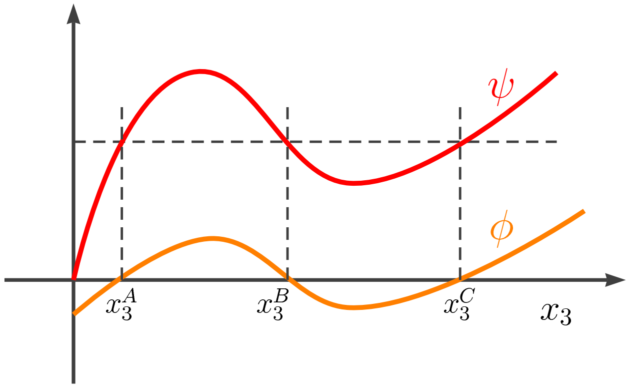

Figure S3: Functions ϕ and ψ

($c_{13}(0) > 0$, according to our assumptions). For large values of x_3, both $\phi(x_3, u)$ and $\psi(x_3)$ asymptotically become linear positive functions of x_3. Therefore, $\psi(x_3)$ asymptotically grows up to $+\infty$ and it has a certain number, say $m \geq 0$ of local minima and maxima. In turn, the equality $\psi(x_3) = a_{32}(u)$ is satisfied in at most $m + 1$ points, which correspond to the zeros of $\phi(x_3, u)$. Let $\bar{x}_3^A \leq \bar{x}_3^B \leq \bar{x}_3^C \ldots$ be these points. The equilibrium conditions $c_{13}(x_3) - b_{11}x_1 = 0$ yields $\bar{x}_1^A \leq \bar{x}_1^B \leq \bar{x}_1^C \ldots$ and the equilibrium condition $a_{21}x_1 - b_{22}x_2 = 0$ implies $\bar{x}_2^A \leq \bar{x}_2^B \leq \bar{x}_2^C \ldots$, therefore $\bar{x}^A \leq \bar{x}^B \leq \bar{x}^C \ldots$, componentwise. For either u small enough or u large enough, there is only one equilibrium (i.e. multiple solutions are possible in a finite interval $0 < u_{min} \leq u \leq u_{max}$).

For any given u, $\bar{x}_3^A \leq \bar{x}_3^B \leq \bar{x}_3^C \ldots$ are also solutions of $\phi(x_3, u) = 0$. It is fundamental to notice that that:

a) if the roots are distinct, then the derivative $\partial\phi(x_3, u)/\partial x_3$ has alternate sign at the roots,

$$\frac{\partial\phi(x_3, u)}{\partial x_3}(\bar{x}_3^A) > 0, \quad \frac{\partial\phi(x_3, u)}{\partial x_3}(\bar{x}_3^B) < 0, \quad \frac{\partial\phi(x_3, u)}{\partial x_3}(\bar{x}_3^C) > 0,$$

b) Corresponding to any equilibrium $\bar{x}_3$, the quantity $a_{32}(u) - b_{32}(\bar{x}_3)$ is positive.

To prove alternate stability–instability consider the Jacoban computed in any equilibrium $\bar{x}$ along with its sign pattern:

$$A = \begin{bmatrix} -b_{11} & 0 & c'_{13}(\bar{x}_3) \\ a_{21} & -b_{22} & 0 \\ 0 & \underbrace{(a_{32}(u) - b_{32}(\bar{x}_3))}_{>0} & -\underbrace{(b_{33} + b'_{32}(\bar{x}_3)\bar{x}_2)}_{>0} \end{bmatrix} \approx \begin{bmatrix} - & 0 & + \\ + & - & 0 \\ 0 & + & - \end{bmatrix},$$

where $\approx$ denotes the sign pattern. In view of b) and the fact that $c'_{13}(x_3) > 0$, A is a Metzler matrix (i.e. its non–diagonal entries are non-negative). It is known that the dominant eigenvalue of a Metzler matrix (having the largest real part) is real. Therefore the system is stable if and only if there are no real

nonnegative eigenvalues. The existence of real nonnegative eigenvalues is equivalent to the existence of non–positive coefficients in the characteristic polynomial. The latter can be derived as follows:

$$p(s) = (s + b_{11})(s + b_{22})(s + (b_{33} + b'_{32}(x_3)x_2)) + \underbrace{(c'_{13}(x_3)(b_{32}(x_3) - a_{32}(u))a_{21})}_{<0}.$$

It is apparent that all the coefficients but the known term are positive. Therefore the stability is equivalent to the positivity of $p_0 = p(0)$. Another round of tedious computations, where we substitute $x_2 = \varphi(x_3) = a_{21}c_{13}(x_3)/(b_{11}b_{22})$ and $\varphi'(x_3) = a_{21}c'_{13}(x_3)/(b_{11}b_{22})$, yields

$$p_0 = b_{11}b_{22}\left(b_{33} + b'_{32}(x_3)\varphi(x_3) + \varphi'(x_3)(b_{32}(x_3) - a_{32}(u))\right) = \frac{\partial \phi(x_3, u)}{\partial x_3}.$$

In view of point a), we proved that the system presents partially ordered equilibria which are alternatively stable–unstable. Note that in the case of a root of multiplicity greater than one, we get $p_0 = b_{11}b_{22}\frac{\partial \phi(x_3,u)}{\partial x_3} = 0$, which means that the system has a zero eigenvalue hence is unstable. If the equilibrium is unique, it is stable.

We now need to prove the last part of this Theorem, namely that the positive and negative cones defined for each equilibrium point are positively invariant. Consider any equilibrium point $\bar{x}$ and the positive cone $x_i \geq \bar{x}_i$, and focus on the face $x_1 = \bar{x}_1$:

$$\dot{x}_1 = c_{13}(x_3) - b_{11}\bar{x}_1 - (c_{13}(\bar{x}_3) - b_{11}\bar{x}_1) = c_{13}(x_3) - c_{13}(\bar{x}_3) \geq 0,$$

where we subtracted the null term $c_{13}(\bar{x}_3) - b_{11}\bar{x}_1$; this inequality holds true in view of the monotonicity of c_{13}. On the face $x_2 = \bar{x}_2$, subtracting the null term $a_{21}\bar{x}_1 - b_{22}\bar{x}_2$, we get

$$\dot{x}_1 = a_{21}x_1 - b_{22}\bar{x}_2 - (a_{21}\bar{x}_1 - b_{22}\bar{x}_2) = a_{21}(x_1 - \bar{x}_1) \geq 0.$$

Finally on the face $x_3 - \bar{x}_3$ we have:

$$\begin{aligned}\dot{x}_3 =& a_{32}(u)x_2 - b_{32}(\bar{x}_3)x_2 - b_{33}\bar{x}_3 - (a_{32}(u)\bar{x}_2 - b_{32}(\bar{x}_3)\bar{x}_2 - b_{33}\bar{x}_3) \\ =& [a_{32}(u) - b_{32}(\bar{x}_3)](x_2 - \bar{x}_2) \geq 0,\end{aligned}$$

where we used again $a_{32}(u)\bar{x}_2 - b_{32}(\bar{x}_3)\bar{x}_2 - b_{33}\bar{x}_3 = 0$. According to Nagumo's theorem, the cone $x_i \geq \bar{x}_i$ is invariant. Reversing all the inequalities, we have that the opposite cone $x_i \leq \bar{x}_i$ is also invariant.

□

S–IV Proof of Proposition 6 (equilibria of the MAPK pathway)

According to (20), in the main text, we can model the MAPK network dynamics in the reduced form:

$$
\begin{aligned}
\dot{x}_1 &= \mu\, a_{17}(x_1)x_7 + c_{10} - b_{11}(x_1)x_1, \\
\dot{x}_2 &= -b_{21}(x_2)x_1 + c_{23}(k - x_2 - x_4), \\
\dot{x}_4 &= a_{41}(k - x_2 - x_4)x_1 - b_{44}(x_4)x_4, \\
\dot{x}_5 &= -b_{54}(x_5)x_4 + c_{56}(h - x_5 - x_7), \\
\dot{x}_7 &= a_{74}(h - x_5 - x_7)x_4 - b_{77}(x_7)x_7.
\end{aligned} \tag{S5}
$$

Recall that mass conservation yields $x_3 = MAP2K_{tot} - x_2 - x_4$, and $x_6 = MAPK_{tot} - x_5 - x_7$.

Proposition 6: For $\mu = 0$ the system admits a unique globally asymptotically stable equilibrium. For $\mu > 0$, the system may have multiple equilibria, for specific choices of the involved functions a, b, c. For $\mu > 0$ suitably large and $a_{17}(x_1)$ lower bounded by a positive number, then the system has no equilibria. For $\mu > 0$ suitably bounded and $a_{17}(x_1)$ increasing, or non-decreasing, and bounded, if several simple[1] equilibria exist, then such equilibria are alternatively stable and unstable. In the special case of three equilibria, then the system is bistable. For $\mu > 0$ suitably bounded and $a_{17}(x_1)$ increasing asymptotically unbounded, then the number of equilibria is necessarily even (typically 0 or 2). Moreover, if we assume that there exists $\mu^* > 0$ such that the system admits two distinct equilibria for any $0 < \mu \leq \mu^*$, then one is stable, while the other is unstable.

Proof: To prove the first part of this proposition, let $\mu = 0$. Then $x_1(t)$ robustly converges to $\bar{x}_1$ such that $b_{11}(\bar{x}_1)\bar{x}_1 = c_{10}$.

Then let $\bar{x}_1$ be fixed and consider the second subsystem Σ_{24} associated with x_2 and x_4. Its steady–state conditions are given by

$$\phi_1(\bar{x}_1, x_2, x_4) \doteq -b_{21}(x_2)\bar{x}_1 + c_{23}(k - x_2 - x_4) = 0, \tag{S6}$$

$$\phi_2(\bar{x}_1, x_2, x_4) \doteq a_{41}(k - x_2 - x_4)x_1 - b_{44}(x_4)x_4 = 0. \tag{S7}$$

As a preliminary step, note that mass conservation allows us to write $x_2 + x_4 \leq k$, where $k = MAP2K_{tot}$. Therefore, the triangle $x_2 \geq 0$, $x_4 \geq 0$ and $x_2 + x_4 \leq k$ (OMN in Figure S4) is invariant. This can be verified by checking the properties of the MN barrier (the system is positive, so MO and ON need not be

[1] i.e. the nullclines have no common tangent lines

checked). We have, for $x_2 + x_4 = k$

$$\dot{x}_4 + \dot{x}_2 = -b_{21}(x_2) - b_{44}(x_4)x_4 < 0.$$

so OMN is positively invariant. Thus there exists an equilibrium point $P = (\bar{x}_2, \bar{x}_4)$, as shown in

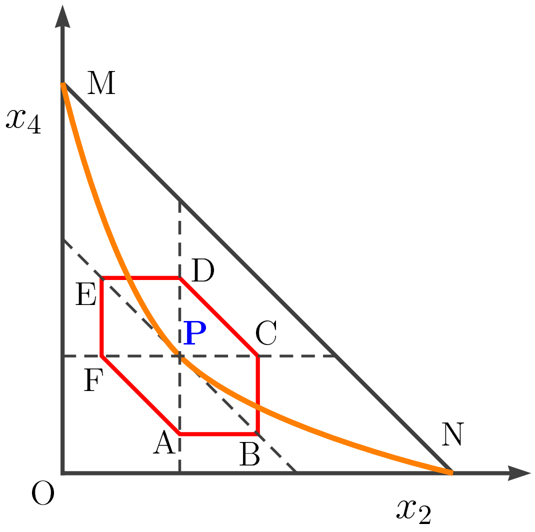

Figure S4: The x_2–x_4 subsystem

Figure S4. This equilibrium is asymptotically stable. We prove this statement by considering the non–smooth piecewise–linear Lyapunov function

$$V(x_2, x_4) = \max\{|x_2 - \bar{x}_2|, |x_4 - \bar{x}_4|, |(x_2 - \bar{x}_2) + (x_4 - \bar{x}_4)|\},$$

whose level surfaces are depicted in red in Figure S4. Note that the red polygon can be divided in six sectors, defined by segments FC and AD, which are parallel to the axes, and EB, which has a -45° slope (the same slope of MN). In each sector, $V(x_2, x_4)$ is equal to the maximum of the three components defined above. Specifically, $V = x_2 - \bar{x}_2$ in BPC and $V = -(x_2 - \bar{x}_2)$ in EPF, $V = x_4 - \bar{x}_4$ in DPE and $V = -(x_4 - \bar{x}_4)$ in APB, $V = (x_2 - \bar{x}_2) + (x_4 - \bar{x}_4)$ in CDP, and the opposite in FPA. We will now look for structural conditions that link the properties of V to the six sectors we defined. First, let us rewrite the dynamics of x_2 and x_4 by adding the zero terms derived from the equilibrium conditions:

$$\begin{aligned}
\dot{x}_2 &= \underbrace{(b_{21}(\bar{x}_2) - b_{21}(x_2))\bar{x}_1}_{\doteq T_{22}} + \underbrace{(c_{23}(k - x_2 - x_4) - c_{23}(k - \bar{x}_2 - \bar{x}_4))}_{\doteq T_{24}}, \\
\dot{x}_4 &= \underbrace{(a_{41}(k - x_2 - x_4) - a_{41}(k - \bar{x}_2 - \bar{x}_4))\bar{x}_1}_{\doteq T_{42}} + \underbrace{(b_{44}(\bar{x}_4)\bar{x}_4 - b_{44}(x_4)x_4)}_{\doteq T_{44}}.
\end{aligned}$$

The terms T_{22}, T_{24}, T_{42}, T_{44}, satisfy the following sign rules: T_{24} and T_{42} are positive below the line EB and negative above EB; T_{22} is negative on the right of line AD and positive on the left of EB. Finally, T_{44} is negative above line FC and negative below. We can thus derive the following conditions:

i) In sector APB, we have $V = -(x_4 - \bar{x}_4)$, and $\dot{V} = -\dot{x}_4 = -T_{42} - T_{44} < 0$ (in the opposite sector, DPE, $V = (\bar{x}_4 - x_4)$ and $\dot{V} = \dot{x}_4 = T_{42} + T_{44} < 0$);

ii) In sector BPC, we have $V = (x_2 - \bar{x}_2)$ so $\dot{V} = \dot{x}_2 = T_{22} + T_{24} < 0$ (in the opposite sector EPF, $V = -(x_2 - \bar{x}_2)$ so $\dot{V} = -\dot{x}_4 = -T_{42} - T_{44} < 0$);

iii) In sector CPD, $V = (x_2 - \bar{x}_2) + (x_4 - \bar{x}_4)$. Then $\dot{V} = \dot{x}_4 + \dot{x}_2 = T_{22} + T_{24} + T_{42} + T_{44} < 0$ (in the opposite sector, FPA, we have $V = -(x_2 - \bar{x}_2) - (x_4 - \bar{x}_4)$, then $\dot{V} = -\dot{x}_4 - \dot{x}_2 = -T_{22} - T_{24} - T_{42} - T_{44} < 0$).

Conditions *i)*–*iii)* are sufficient to state that $D^+V < 0$ for $(x_2, x_4) \neq (\bar{x}_2, \bar{x}_4)$, hence $(x_2(t), x_4(t)) \to (\bar{x}_2, \bar{x}_4)$. In turn this implies the uniqueness of the equilibrium point for any fixed $\bar{x}_1$, so that $(\bar{x}_2, \bar{x}_4)$ are functions of $\bar{x}_1$: $\bar{x}_2 = \bar{x}_2(\bar{x}_1)$ and $\bar{x}_4 = \bar{x}_4(\bar{x}_1)$.
From (S6)(S7) we see that for $\bar{x}_1 \to 0$, $(\bar{x}_2, \bar{x}_4) = (k, 0)$ and for $\bar{x}_1 \to \infty$, $(\bar{x}_2, \bar{x}_4) = (0, k)$. Note that in view of the constraint $x_2(t) + x_3(t) + x_4(t) = k$, the dynamics of x_3 will vanish in both cases. For intermediate values of x_1, the points $(\bar{x}_2(\bar{x}_1), \bar{x}_4(\bar{x}_1))$ describe the orange curve NPM depicted in Figure S4. For reasons that will be clear later, we further explore this point. From the implicit function theorem, since $(\bar{x}_2, \bar{x}_4)$ are derived implicitly from (S6) and (S7) we have the following expression for the derivatives[2]

$$\begin{aligned}
\frac{d}{dx_1}\begin{bmatrix} x_2 \\ x_4 \end{bmatrix} &= -\begin{bmatrix} -(b'_{21}x_1 + c'_{23}) & -c'_{23} \\ -a'_{41}x_1 & -(a'_{41}x_1 + (b_{44}(x_4)x_4)') \end{bmatrix}^{-1} \begin{bmatrix} -b_{21}(x_2) \\ a_{41}(k - \bar{x}_2 - \bar{x}_4) \end{bmatrix} \\
&= -\frac{1}{\Delta_{24}} \begin{bmatrix} -(a'_{41}x_1 + (b_{44}(x_4)x_4)') + c'_{23}) & +c'_{23} \\ +a'_{41}x_1 & -(b'_{21}x_1 + c'_{23}) \end{bmatrix} \begin{bmatrix} -b_{21}(x_2) \\ a_{41}(k - \bar{x}_2 - \bar{x}_4) \end{bmatrix},
\end{aligned}$$

where Δ_{24} is the determinant of the inverted matrix. From our assumptions, it can be verified that $\Delta_{24} > 0$. Then the following relation holds for steady–state values:

$$\begin{aligned}
\frac{dx_2}{dx_1} &= -\frac{(a'_{41}x_1 + (b_{44}(x_4)x_4)')b_{21}(x_2) + c'_{23}a_{41}}{\Delta_{24}} < 0, \\
\frac{dx_4}{dx_1} &= \frac{a'_{41}x_1 b_{21} + (b'_{21}x_1 + c'_{23})a_{41}}{\Delta_{24}} > 0.
\end{aligned}$$

This implies that the steady state $\bar{x}_2$ decreases when $\bar{x}_1$ increases, while $\bar{x}_4$ increases when $\bar{x}_1$ increases.

[2] if $y = F(x)$ is derived from $\Phi(x, y) = 0$, then $dy/dx = -[\partial\Phi/\partial y]^{-1}\partial\Phi/\partial x$, where $[\partial\Phi/\partial y]$ is the Jacobian

The *exact same analysis* can be repeated for for the subsystem Σ_{57}, with input x_4. In particular, the steady state $\bar{x}_5$ is a decreasing with $\bar{x}_4$ while $\bar{x}_7$ increasing. Furthermore, $(x_5(t), x_7(t)) \to (\bar{x}_5, \bar{x}_7)$, for fixed $\bar{x}_4$. With standard Lyapunov arguments, considering the boundedness of all variables, we can prove that $x_1 \to \bar{x}_1$ implies $(x_2(t), x_4(t)) \to (\bar{x}_2, \bar{x}_4)$ which, in turn, implies $(x_5(t), x_7(t)) \to (\bar{x}_5, \bar{x}_7)$, hence stability holds. The following facts are worth pointing out:

a) all the formulas so far derived are valid if we replace the index 2 by 5 and 4 by 7. In particular we have that for steady–state values

$$\frac{dx_7}{dx_4} = \frac{a'_{74}x_4b_{54} + (b'_{54}x_4 + c'_{56})a_{74}}{\Delta_{57}} > 0.$$

b) for a steady state value $\bar{x}_1$, the corresponding value of $\bar{x}_7$ is achieved by a compound function $\varphi : \bar{x}_1 \to \bar{x}_4 \to \bar{x}_7$, namely $\bar{x}_7 = \varphi(\bar{x}_1)$. This function is increasing (both its components are such) and its derivative is

$$\varphi'(x_1) = \frac{dx_7}{dx_4}\frac{dx_4}{dx_1} = \frac{(a'_{41}x_1b_{21} + (b'_{21}x_1 + c'_{23})a_{41})}{\Delta_{24}} \frac{(a'_{74}x_4b_{54} + (b'_{54}x_4 + c'_{56})a_{74})}{\Delta_{57}} > 0. \tag{S8}$$

Biologically, this means that this cascade model indeed transmits the input signal by increasing the concentration of the active, doubly–phosphorylated species MAP2K–PP and MAPK–PP.

Let us consider now the positive feedback, $\mu > 0$. At steady state $\dot{x}_1 = 0$, $\mu\ a_{17}(x_1)x_7 + c_{10} - b_{11}(x_1)x_1 = 0$, and in view of $x_7 = \varphi(x_1)$, we have the steady state equation:

$$\mu a_{17}(x_1)\varphi(x_1) + c_{10} = b_{11}(x_1)x_1.$$

The two functions appearing of both sides are depicted in Figure S5. It is apparent that, for μ small enough, there is an even number of intersections between the two curves. For large values there are no intersections because function $a_{17}(x_1)\varphi(x_1)$ is strictly increasing and $b_{11}(x_1)x_1$ is bounded.

For convenience we study first the case in which $\mu a_{17}(x_1)$ grows unbounded and there are only two simple roots. Write the steady–state equation equivalently as

$$\psi(x_1) \doteq b_{11}(x_1)x_1 - \mu a_{17}(x_1)\varphi(x_1) - c_{10} = 0,$$

(the blue curve in Figure S5) and denote by $\bar{x}_1^A$ and $\bar{x}_1^B$ its roots. In such positions we have $\psi'(\bar{x}_1^A) > 0$ and $\psi'(\bar{x}_1^B) < 0$, respectively, a condition we will use soon to study the stability of the two equilibria. To this aim let us consider the Jacobian of the reduced model of the cascade, where the linearized system variables are $\delta x_1 = x_i - \bar{x}_i$, $i = 1, 2, 4, 5, 7$:

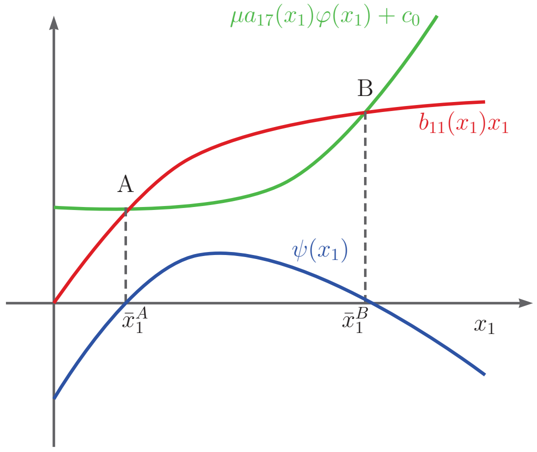

Figure S5: Functions $\mu a_{17}(x_1)\varphi(x_1) + c_{10}$, $b_{11}(x_1)x_1$ and their difference $\psi(x_1)$.

$$J = \begin{bmatrix} -(b_{11}x_1)' + \mu a'_{17}x_7 & 0 & 0 & 0 & \mu a_{17} \\ -b_{21} & -[b'_{21}x_1 + c'_{23}] & -c'_{23} & 0 & 0 \\ a_{41} & -a'_{41}x_1 & -[a'_{41}x_1 + (b_{44}x_4)'] & 0 & 0 \\ 0 & 0 & -b_{54} & -[b'_{54}x_4 + c'_{56}] & -c'_{56} \\ 0 & 0 & a_{74} & -a'_{74}x_4 & -[a'_{74}x_4 + (b_{77}x_7)'] \end{bmatrix}. \quad \text{(S9)}$$

This is not a Metzler matrix, but it can be reduced to the Metzler form by the similarity transformation $T^{-1}JT$ with $T = diag\{1, -1, 1, -1, 1\}$, in brief, if we change sign to δx_2 and δx_5. A necessary condition for stability is that the constant term of the characteristic polynomial is positive, i.e. $p_0 = det(-J) > 0$. We get

$$p_0 = ((b_{11}x_1)' - \mu a'_{17}x_7)\Delta_{24}\Delta_{57} - \mu a_{17}(a'_{41}x_1b_{21} + (b'_{21}x_1 + c'_{23})a_{41})\ (a'_{74}x_4b_{54} + (b'_{54}x_4 + c'_{56})a_{74}),$$

where we recall that Δ_{24} and Δ_{57} are the determinants of the subsystems Σ_{24} and Σ_{57}. Divide the above expression by the positive terms Δ_{24} and Δ_{57}; by noticing that the terms in square brackets are identical to those in (S8) and by taking into account that $x_7 = \varphi(x_1)$ at steady state, we obtain

$$\frac{p_0}{\Delta_{24}\Delta_{57}} = (b_{11}x_1)' - \mu a'_{17}\varphi(x_1) - \mu a_{17}\varphi'(x_1) = (b_{11}x_1)' - \frac{d}{dx_1}[\mu a_{17}\varphi(x_1)] = \psi'(x_1).$$

Since $\psi'(\bar{x}_1^B) < 0$ we conclude that point B is unstable for $0 < \mu \leq \mu^*$.

To show stability of point A we must remember that the opposite condition holds $\psi'(\bar{x}_1^A) > 0$, $0 < \mu \leq \mu^*$. Since the eigenvalues depend continuously on the system parameters, we have stability for μ small enough. Moreover, since the Jacobian is similar to a Metzler matrix, it has a real dominant eigenvalue and thus transition to instability (if any) for μ increasing must occur corresponding to $\mu_0 < \mu^*$ in which J has a zero eigenvalue, which implies $p_0 = 0$, and therefore the contradiction $\psi'(\bar{x}_1^A) = 0$. Hence A must be stable.

Alternate stability of the equilibria for $a'_{17} \geq 0$ (with a_{17} bounded or constant) can now be proved by using the previous material. To this aim we show that any matrix having the form (S9) is stable if and only if its characteristic polynomial is positive in $s = 0$. Such a polynomial is given by

$$p(s, \mu) = q(s) - \mu \left[a'_{17} x_7 r(s) + a_{17} m(s)\right],$$

where $r(s)$ and $m(s)$ are polynomials with positive coefficients:

$$r(s) = det \begin{pmatrix} s + [b'_{21}x_1 + c'_{23}] & c'_{23} \\ a'_{41}x_1 & s + [a'_{41}x_1 + (b_{44}x_4)'] \end{pmatrix} det \begin{pmatrix} s + [b'_{54}x_4 + c'_{56}] & c'_{56} \\ a'_{74}x_4 & s + [a'_{74}x_4 + (b_{77}x_7)'] \end{pmatrix},$$

$$m(s) = det \begin{pmatrix} b_{21} & s + [b'_{21}x_1 + c'_{23}] \\ -a_{41} & a'_{41}x_1 \end{pmatrix} det \begin{pmatrix} b_{54} & s + [b'_{54}x_1 + c'_{56}] \\ -a_{74} & a'_{74}x_4 \end{pmatrix},$$

while

$$q(s) = (s + (b_{11}x_1)')r(s).$$

Matrix J is similar to a Metzler matrix for any $\mu \geq 0$, then it has a dominant real eigenvalue. For $\mu = 0$, $p(s) = q(s)$ has positive coefficients, thus it has no positive or zero real roots and therefore all its roots have negative real part. Define the following value [3]

$$\mu^* = \inf\{\mu > 0 : p(s, \mu) \text{ has unstable roots}\}.$$

Since the dominant eigenvalue is real, the polynomial at the stability boundary, namely $p(s, \mu^*)$, has a root in zero, say $p(0, \mu^*) = 0$. On the other hand, its constant term is $p_0 = q_0 - \mu[a'_{17}x_7 r(0) + a_{17}m(0)]$, which is obviously negative for $\mu > \mu^*$. Therefore the necessary and sufficient condition for stability is

$$p_0 = p(0, \mu) = q_0 - \mu[a'_{17}x_7 r(0) + a_{17}m(0)] > 0.$$

To conclude the proof, we need only to reconsider the previous expression

$$\frac{p_0}{\Delta_{24}\Delta_{57}} = \psi'(x_1),$$

to conclude that stability of the equilibrium depends only on the type of intersection. Since in the first intersection point A in Fig. S6 we have $\psi'(x_1^A) > 0$ the first equilibrium is stable and the remaining, alternatively, stable–unstable. The three–point case is depicted in Fig. S6 which represents a bistable situation. □

[3] The parametric study which follows is not affected by the fact that the intersection point is a function of μ, and it is generically valid for any matrix of the form of J.

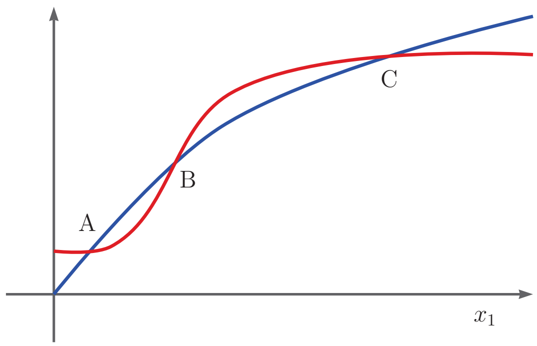

Figure S6: Functions $\mu a_{17}(x_1)\varphi(x_1) + c_{10}$ (red) and $b_{11}(x_1)x_1$ (blue): the points A and C are stable while B is unstable

References

1. Slotine J: *Applied Nonlinear Control*. New Jersey: Prentice Hall 1991.
